# Endothelin Receptors and Their Antagonists^☆^^☆☆^

**DOI:** 10.1016/j.semnephrol.2015.02.002

**Published:** 2015-03

**Authors:** Janet J. Maguire, Anthony P. Davenport

**Affiliations:** Clinical Pharmacology Unit, University of Cambridge, Addenbrooke׳s Hospital, Cambridge, United Kingdom

**Keywords:** Ambrisentan, antagonist, bosentan, endothelin-1, macitentan, sitaxentan

## Abstract

All three members of the endothelin (ET) family of peptides, ET-1, ET-2, and ET-3, are expressed in the human kidney, with ET-1 being the predominant isoform. ET-1 and ET-2 bind to two G-protein–coupled receptors, ET_A_ and ET_B_, whereas at physiological concentrations ET-3 has little affinity for the ET_A_ receptor. The human kidney is unusual among the peripheral organs in expressing a high density of ET_B_. The renal vascular endothelium only expresses the ET_B_ subtype and ET-1 acts in an autocrine or paracrine manner to release vasodilators. Endothelial ET_B_ in kidney, as well as liver and lungs, also has a critical role in scavenging ET-1 from the plasma. The third major function is ET-1 activation of ET_B_ in in the nephron to reduce salt and water re-absorption. In contrast, ET_A_ predominate on smooth muscle, causing vasoconstriction and mediating many of the pathophysiological actions of ET-1. The role of the two receptors has been delineated using highly selective ET_A_ (BQ123, TAK-044) and ET_B_ (BQ788) peptide antagonists. Nonpeptide antagonists, bosentan, macitentan, and ambrisentan, that are either mixed ET_A_/ET_B_ antagonists or display ET_A_ selectivity, have been approved for clinical use but to date are limited to pulmonary hypertension. Ambrisentan is in clinical trials in patients with type 2 diabetic nephropathy. This review summarizes ET-receptor antagonism in the human kidney, and considers the relative merits of selective versus nonselective antagonism in renal disease.

All three members of the endothelin (ET) family of peptides, ET-1, ET-2, and ET-3, are expressed in the human kidney, although ET-1 is the predominant isoform. ET-1 and ET-2 bind to two G-protein–coupled receptors, ET_A_ and ET_B_, whereas at physiological concentrations ET-3 has little affinity for the ET_A_ receptor. The endothelin receptors are members of the Family A G-protein–coupled receptors, a class of proteins that has been exploited very successfully as targets for the development of drugs.

The human kidney is unusual among the peripheral organs in expressing a high density of ET_B_. The renal vascular endothelium only expresses the ET_B_ subtype and ET-1 acts in an autocrine or paracrine manner to release vasodilators. Endothelial ET_B_ in kidney, as well as liver and lungs, has a critical role in scavenging ET-1 from the plasma. The third major function is for ET-1 activation of ET_B_ in medullary epithelial cells to reduce salt and water reabsorption. ET_A_ predominate on the vasculature to cause vasoconstriction. The pathophysiological actions of ET-1 are mediated mainly via the ET_A_ subtype. The role of the two subtypes has been delineated in preclinical and acute experimental studies using highly selective ET_A_ (including BQ123, TAK-044) and ET_B_ (BQ788) peptide antagonists. Three nonpeptide antagonists, bosentan, macitentan, and ambrisentan, that are either mixed ET_A_/ET_B_ antagonists or display ET_A_ selectivity, have been approved for clinical use, primarily in pulmonary arterial hypertension.

In renal pathophysiological conditions ET-1 contributes to vascular remodeling, proliferation of mesangial cells, and extracellular matrix production, mainly through binding to ET_A_. Beneficial actions of ET-1 on sodium and water regulation mainly are ET_B_-mediated. These findings suggest an ET_A_-selective antagonist would have a therapeutic advantage over a mixed antagonist in renal disease. Acute studies directly comparing mixed and selective peptide antagonists suggest selective ET_A_ blockade, however, sparing ET_B_ may be beneficial. However, this was balanced by a greater prevalence of side effects for small-molecule, orally active ET_A_ antagonists compared with mixed antagonists, although the latter also have their limitations. The ET signaling pathway in the kidney remains a promising clinical target for receptor antagonism, which may be realized by the next generation of antagonists.

## ET Receptors

**Figure 1:**
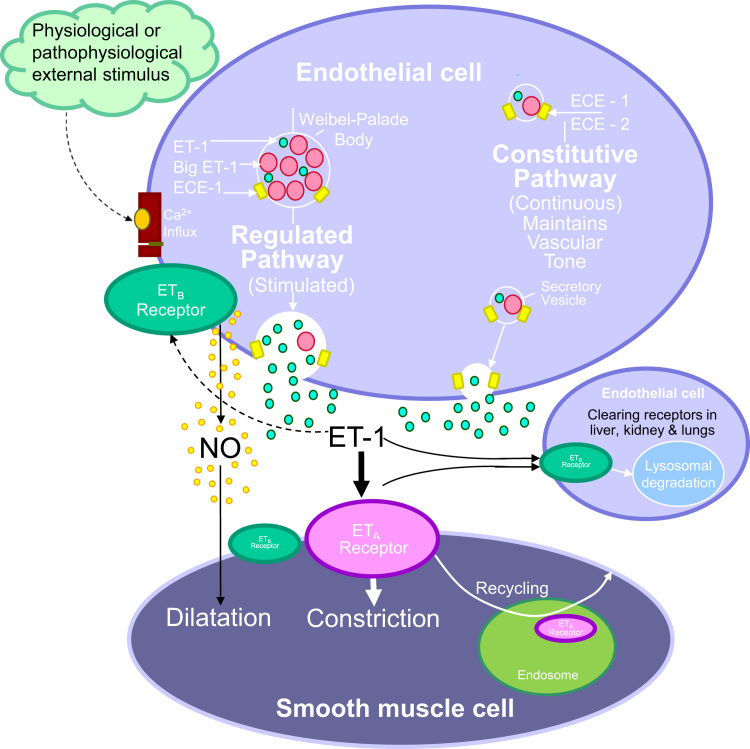
Model of the ET-1 signaling pathway in the renal vasculature. The primary source of ET-1 production is the vascular endothelium, although it also is produced by other cell types in the kidney including epithelial cells. ET-1 is synthesized within the secretory vesicles of the constitutive pathway. Pro–ET-1 is processed to big ET-1 by the action of a furin convertase. Big ET-1 then is transformed to the mature, biologically active peptide ET-1, mainly through the action of ECE-1, although a second related enzyme, ECE-2, also may play a role, particularly under acidic pathophysiological conditions. ET-1 also is released from Weibel-Palade bodies of the regulated pathway in response to external stimuli. ET-1 released abluminally causes the underlying smooth muscle to contract, mainly via ET_A_. The ligand-receptor complex then undergoes internalization to the endosome before recycling of the receptor to the cell surface. Some smooth muscle cells from specific vascular beds express a low density of ET_B_, but ET_A_ antagonists are able to fully reverse an established ET-1 constrictor response, implying a minimal contribution. Binding to endothelial ET_B_ elicits an opposing vasodilatation via the release of relaxing factors as well as removal of ET-1 from the circulation by internalization to the lysosome and degradation.

The ET family comprises three isoforms, ET-1, ET-2, and ET-3.^1,2^ Although messenger RNA encoding all three has been detected in human kidney, ET-1 is the predominant intrarenal isoform.^3^ ETs interact with two distinct G-protein–coupled receptors, ET_A_^4^ and ET_B_^5^ (Fig. 1), which were identified 2 years after the discovery of the endogenous peptides in 1988. They are both class A, G-protein–coupled receptors; this class is the target of nearly half of currently available medicines. This has resulted from well-developed medicinal chemistry strategies and high-throughput screening programs to identify small-molecule drugs, stimulating considerable effort to discover ET-receptor antagonists. The initial clue to the existence of two subtypes and the key to classifying the receptors was that ET-1 and ET-2 are equipotent at the ET_A_ subtype whereas ET-3 shows at least 100-fold lower potency and at physiological concentration ET-3 is unlikely to activate this subtype (Table 1). All three ETs bind to ET_B_ with similar affinity.^6,7^ This review focuses on the role of ET receptors in the human kidney and considers the clinical pharmacology of ET antagonists that have been used to block these receptors. The effects of ET-1 on the kidney are complex and more detailed information can be found in reviews on renal endothelin physiology^8^ and pathology,^9^ and the pharmacology of the endothelin signaling pathway.^10,11^

## No Evidence for Further ET-Receptor Subtypes

Further receptor subclassifications have been proposed including suggestions that ET_B_ could be subdivided into ET_B1_, present on endothelial cells, and ET_B2_ on smooth muscle cells, but there currently is no evidence that the receptors expressed by these two cell types can be distinguished pharmacologically.^10,11^ ET-receptor antagonists have not been successful in certain conditions such as heart failure,^12^ perhaps implying that ETs may mediate their actions via previously unsuspected receptors; however, this is unlikely. Following the sequencing of the human genome, it is accepted that all genes that potentially encode a G-protein–coupled receptor have been identified and currently are classified as ‘orphan’ to indicate that their endogenous ligand is not yet known.^13,14^ These remaining orphan receptors (approximately 80) have been screened against more than 20 ET peptides (including all three endogenous isoforms and their corresponding big ET precursors, C-terminal metabolites, the ET_A_ antagonist BQ123, and the ET_B_ agonist BQ3020) without detectable binding. The screen also included two of the most closely related orphan receptors to ET_A_ and ET_B_, GPR37 (also known as *endothelin-receptor type B-like receptor* or *Parkin-associated endothelin receptor-like receptor*) and its related receptor GPR37L1. Two neuropeptides, prosaptide and prosaposin, that are structurally distinct from the ETs have been suggested to be the endogenous ligands for GPR37 and GPR37L1.^15^

## Peptide Agonists

Experimental medicine studies in volunteers mainly use ET-1 that is equipotent for ET_A_ and ET_B_ (Table 1). ET-3, which is modestly selective for ET_B_,^7^ also has been used but greater ET_B_ selectivity is shown by sarafotoxin S6c, one of the isoforms originally identified from snake venom.^16^ IRL1620 (Suc-[Glu^9^,Ala^11,15^]-endothelin-1_8-21_)^17^ is a truncated linear analogue in which the N-terminus has an N-succinyl modification, reducing metabolism by nonspecific peptidases. It was developed as an ET_B_ agonist but now is used in clinical trials as a potential vasodilator in the delivery of anticancer agents and in neuroprotection where it is known as SPI-1620 (licensed by Spectrum Pharmaceuticals, Henderson, NV). The second widely used ET_B_ agonist is BQ3020 ([Ala^11,15^]Ac-ET-l_6-21_),^18^ however, this compound has not been used clinically.

## Peptide Antagonists

The first endothelin-receptor antagonists to be discovered were from natural product screening, compound libraries, or drug design based on the structure of the endogenous ET peptides (Table 1). The most widely used, according to the number of published articles, is the cyclic pentapeptide BQ-123 (D-Asp-L-Pro-D-Val-L-Leu-D-Trp-) (Ihara et al^19^), based on peptides isolated from *Streptomyces misakiensis*, a highly selective competitive ET_A_ antagonist with low nanomolar affinity for the receptor. The second most widely used is FR 139317 (N-[(hexahydro-1-azepinyl)carbonyl]L-Leu[1-Me]D-Trp-3 [2-pyridyl]-D-Ala),^20^ a linear tripeptide. These are both highly ET_A_ selective for human (as well as rodent) ET receptors and at concentrations used in experimental medicine or in vivo animal experiments are likely to block only the ET_A_ receptor; data from these studies can be interpreted with confidence. TAK-044 is a cyclic hexapeptide also isolated from *S misakiensis* with a more modest degree of ET_A_ selectivity.^21^ BQ788 (N-[([2R,6S]-2,6-dimethyl-1-piperidinyl)carbonyl]-4-methyl-L-leucyl-N-[(1R)-1-carboxylatopentyl]-1-[methoxycarbonyl]-D-tryptophanamide) is a modified tripeptide developed by structure-activity analysis^22^ and is a selective competitive ET_B_ antagonist (usually showing one to two orders of magnitude selectivity for ET_B_ over ET_A_) in human beings and across species. Because these compounds are all peptides, they have little or no oral bioavailability, require intra-arterial administration, and are metabolized or excreted over comparatively short periods of time. An advantage in their use is that they are soluble and do not bind plasma proteins. Therefore, they are used for short-term, acute investigations in both animal models and in experimental medicine studies.

## ET_A_ Receptors Predominate on Smooth Muscle of Renal Vessels and Mediate Vasoconstriction

A major physiological action of ET-1 is to function as one of the most powerful vasoconstrictors of human blood vessels. As such, ET-1 plays a major role in regulating vascular function in all organ systems, including the kidney (Fig. 1). As in other vessels, ET-1 is thought to be released from endothelial cells lining intrarenal vessels throughout the cortex and medulla. In the human vasculature, including that of the kidney, under normal physiological conditions release of ET-1 from endothelial cells causes sustained vasoconstriction via ET_A_ that predominate on the underlying smooth muscle. Under pathophysiological conditions in which ET-1 is overproduced, vascular cells also may undergo proliferation and contribute to vascular remodeling and the development of renal fibrosis. Figure 1 shows the ratio of the densities of the two receptor subtypes measured by radioligand binding assays with the ET_A_ subtype representing greater than 90% of ET receptors in the smooth muscle layer of all renal vessels studied. This includes the large conduit vessels, the arcuate arteries, and veins at the corticomedullary junction, as well as small intrarenal vessels such as the afferent and efferent vessels of the glomerulus.^23–27^

In a detailed study using human isolated main stem renal arteries and veins in organ baths,^28^ ET-1 was, as expected, a potent vasoconstrictor, with the concentration producing half-maximal response (EC_50_) values of 4 and 1 nmol/L, respectively. In renal artery, ET-3 and the ET_B_ agonist sarafotoxin 6c showed little or no activity up to 300 nmol/L. In veins, some but not all samples responded to ET-3, but this peptide was much less potent than ET-1, consistent with an ET_A_- mediated action. Interestingly, S6c concentration-related contractions were found in some individuals and, although more potent than ET-1, the maximum response was 30% to 60% of that obtained with ET-1. Crucially, however, the ET_A_ antagonist BQ123 fully reversed the ET-1 contractions in both arteries and veins without reducing the maximum agonist response, consistent with a competitive antagonist. Therefore, in renal vessels the endogenous peptides ET-1 and ET-3 appear to mediate vasoconstriction via the ET_A_, indicating that ET_B_-mediated responses in human renal vessels are of little importance. The pharmacology of isolated renal arteries and veins is similar to vessels obtained from other human vascular beds, with ET_A_ antagonists fully reversing an ET-1 response.^29^ This is critical to understanding the importance of selectivity for the two subtypes. Sarafotoxin S6c–induced constrictor responses have been used previously as evidence of significant ET_B_ constrictor responses in human vessels. However, it is not an endogenous ligand and ET-1 responses are fully reversed using ET antagonists. Bohm et al^30^ performed key experimental medicine studies that showed in volunteers in vivo that BQ123 inhibited the ET-1–mediated increase in renal vascular resistance whereas BQ788 (ET_B_ antagonist) potentiated the ET-1 effect, implying a constrictor role for ET_A_ and that ET_B_ clears ET-1 from the plasma. Kaasjager et al^31^ also concluded that the systemic and renal vasoconstrictor effects of ET-1 in human beings are mediated by the ET_A_.

A further unusual feature of ET-1 compared with other vasoconstrictors is that the constrictor response is sustained over a considerable period of time, lasting for several hours or in some cases several days.^32^ Contractions compared with many other vasoconstrictors are slow to wash out, which is consistent with a slow dissociation rate for ET-1 and may contribute to sustained hypertension and/or ET-induced vasospasm associated with pathophysiological conditions such as chronic kidney disease. Importantly, ET antagonists are able to relax ET_A_- mediated vasoconstriction in vessels preconstricted with ET-1^33^ and this may reflect rapid internalization of the ligand receptor complex for recycling to the membrane (Fig. 1). In contrast, binding of ET-1 to ET_B_ in vivo often is not displaced by ET_B_ antagonists,^34^ which is in agreement with ET_B_ being internalized by a different pathway and degraded in the lysosome.

## How Important is the Small Population of ET_B_ Receptors Expressed by Vascular Smooth Muscle?

In some, but not all, human vessels, a small population of ET_B_ (usually <15%) can be measured by ligand binding. Although in some human beings isolated renal vessel responses to high concentrations of ET-3 were detected,^28^ comparison of equipotent concentrations of ET-3 and ET-1 in healthy volunteers found that ET-3 had no effect on blood pressure or renal hemodynamics,^31^ which might have been expected if ET_B_ contributed significantly to a contractile response. Whether the proportion of vascular ET_B_ changes with disease remains controversial and has not been studied in detail in pathophysiological renal tissue. However, detailed studies in vitro in human coronary arteries with atherosclerotic lesions did not show any increase.^35^ In agreement, in experimental medicine studies in both heart failure patients and volunteer controls, selective ET_A_ antagonism (BQ123) caused the expected potent vasodilatation in the peripheral circulation. However, BQ788 caused vasoconstriction in both groups, consistent with blocking endothelial cell ET_B_-mediated vasodilatation, with no evidence of contractile ET_B_.^36,37^

## Other Cell Types Expressing ET_A_ Receptors

ET_A_ have been shown to be present on human and rat podocytes (glomerular epithelial cells) that wrap around the capillaries of the glomerulus within Bowman׳s capsule.^38,39^ Ortmann et al^40^ also detected messenger RNA encoding ET_B_ as well as ET_A_ on human podocytes. However, ET_A_ contribute to podocyte injury through cytoskeleton disruption and apoptosis and only ET_A_ antagonists are effective in preventing podocyte injury. In renal disease, proliferation in mesangial cells, extracellular matrix production, and inflammation^41,42^ are mediated mainly by ET_A_.

## ET_B_ Receptors Predominate in the Kidney and Mediate Beneficial Vasodilatation, Clearing of ET-1 from Plasma, and Natriuresis

**Figure 2:**
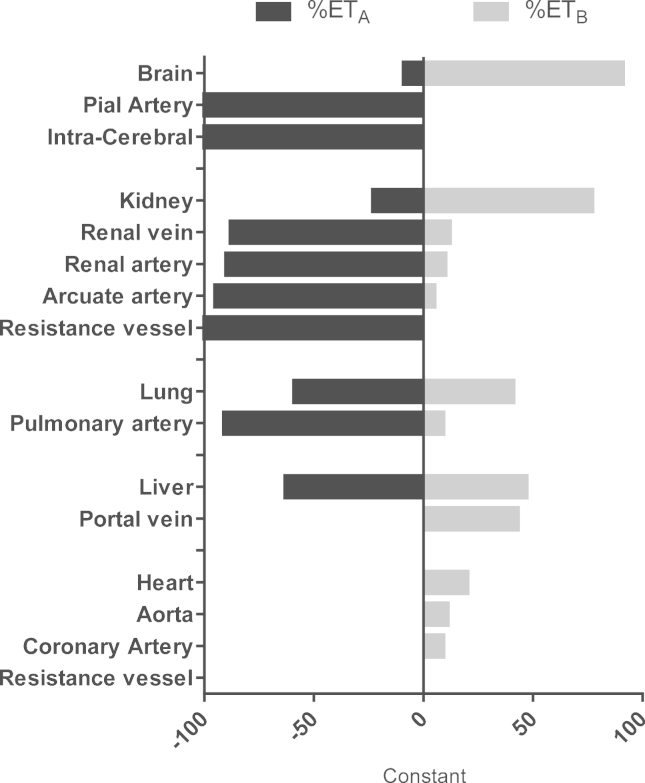
Ratio of the density of human ET_A_ and ET_B_ measured using radioligand binding in the whole organ (brain, kidney, lung, liver, and heart) and measured in the medial smooth muscle layer of the vasculature within each organ. In human beings, kidney, lung, and liver are ET_B_-rich, reflecting the expression of ET_B_ receptors on endothelium of the vasculature and other cell types such as the epithelium. In contrast, in the heart, ET_A_ are the principal subtype reflecting expression on myocytes. In the smooth muscle layer of all human vessels, ET_A_ are more abundant than ET_B_.

In peripheral tissues such as the heart (Fig. 1), ET_A_ are more abundant (>60%) than ET_B_ (Fig. 2). In marked contrast, in the kidney, lungs, and liver this ratio is reversed. Although measurements of receptors within smooth muscle throughout the renal vasculature show a predominance of ET_A_, 70% of the ET receptors in both cortex and medulla in human kidney are ET_B_. ET_B_ predominate, reflecting, at least in part, that these are endothelial cell–rich tissues similar to liver and lungs.^24–26^ Endothelial cells line every vessel wall and have a mass comparable with other endocrine organs. Although ET_B_ also are expressed by other cell types, selective deletion of the endothelial cell ET_B_, leaving ET_B_ on other cells intact, shows that in many organs, including the kidney, liver, and lungs, endothelial cells represent the majority of the receptors.^43^

A consensus has emerged that ET_B_ mediates vasodilatation by the release of endothelium-derived relaxing factors (nitric oxide, prostacyclin, and/or endothelium-derived hyperpolarizing factor), acting as a feedback mechanism to limit the vasoconstrictor action of ET-1. Infusions of ET-1 into the brachial artery of volunteers produces a biphasic response: low doses of ET-1 cause ET_B_-mediated vasodilatation, however, as the concentration increases to higher pathophysiological concentrations, vasodilatation is overwhelmed by ET_A_-mediated constrictor responses. When endothelial dysregulation occurs in renal disease there is a loss of opposing vasodilators, leading to increased vasoconstriction and vasospasm.

Endothelial cell ET_B_ function as scavenging or clearing receptors to remove ET-1 from the circulation,^23,43,44^ particularly by the ET_B_-rich tissues: kidney, lungs, and liver^34^ (Fig. 1). Selectively blocking ET_A_, but not ET_B_, with a low dose of the peptide antagonist TAK-044 infused into volunteers caused no change in measured plasma ET-1 levels. However, a higher dose that blocked both subtypes increased ET-1 levels by more than three-fold as a result of reducing clearing by ET_B_.^45^

In renal circulation, in agreement with other vascular beds in human beings, systemic infusion of ET-1, which activates both receptors, into volunteers increased blood pressure (6 mm Hg), and decreased renal plasma flow, glomerular filtration rate, and sodium excretion rate.^31^ Although BQ123 infused alone did not affect basal arterial blood pressure or renal or splanchnic vascular resistance, the antagonist inhibited the increase in vascular resistance induced by co-infusion of ET-1. In contrast, BQ788 alone caused the opposite effect: increased renal or splanchnic vascular resistance, consistent with blocking endothelial cell–receptor vasodilatation. Second, BQ788 potentiated the ET-1–induced increase in vascular resistance mediated by ET_A_, suggesting that blocking the scavenging receptors modulated plasma ET-1 levels.^30^ Inhibition of tonic nitric oxide production by inhibition of nitric oxide synthase elicits vasoconstriction with an increase in mean arterial pressure and vascular resistance in many organs, including the kidney. Renal and systemic vasoconstriction in volunteers caused by the nitric oxide synthase inhibitor N-nitro-L-arginine methyl ester were attenuated by BQ123, supporting the concept that the balance between endogenous nitric oxide production and ET-1/ET_A_ activity contributes to renal and systemic tone in human beings.^46,47^

The role of ET_B_ clearing receptors has been studied in detail in endothelial cell–specific ET_B_ knock-out mice. In these animals, clearance of an intravenous bolus of labeled ET-1 was reduced significantly compared with wild-type controls. Importantly, functioning ET_B_ were retained on all other cell types such as epithelial cells.^43,48^ Dynamic imaging of rats using positron emission tomography showed that after infusion of ^18^F ET-1, there was remarkably fast clearance of the radioligand from the circulation (plasma-half life (*t*_1/2_) = 0.43 min), with high levels of radioligand accumulated in the kidney, liver, and lung, which rapidly reached equilibrium, and this was maintained for at least 20 minutes. Infusion of BQ788 before injecting ^18^F ET-1 reduced the amount of radioligand visualized in the lung and kidney by 85% and 55%, respectively, consistent with blockade of ET_B_. However, infusion of BQ788 after ^18^F ET-1 did not displace the bound ligand.^34^ This finding is consistent with the internalization of the ligand-receptor complex to the lysosome where ET-1 is thought to be degraded, similar to other peptides, by cathepsin A. In support, cathepsin A knock-out mice showed reduced ET-1 degradation and significantly increased arterial blood pressure.^49^ Inactivation of ET-1 by kidney, liver, and lungs may be particularly important for ET-1 because it is structurally unusual compared with other vasoactive peptides, possessing two disulfide bridges that confer resistance to degradation by nonspecific peptidases.

ET-1 promotes diuresis and natriuresis via ET_B_ located on epithelial cells throughout the tubular epithelium, particularly the inner medullary collecting duct cells.^50^ Deletion of ET_B_, but not ET_A_, leads to salt-sensitive hypertension.^51^ In agreement, the effects of three doses of BQ-123 (0.1, 0.2, and 0.3 mg/kg) on renal hemodynamics, tubular function, and vasoactive hormones were measured in volunteers in a randomized, placebo-controlled, double-blind, dose-response study. The main effect was a dose-dependent increase in renal sodium excretion despite stimulation of the renin-angiotensin system as evidenced by an increase in angiotensin II levels, whereas there was little effect on atrial and brain natriuretic peptides or vasopressin.^52^

Goddard et al^53^ elegantly showed that ET_A_ antagonism by BQ123 and angiotensin-converting enzyme (ACE) inhibition using enalapril were synergistic in reducing mean arterial pressure in volunteers. However, BQ-123 increased renal blood flow, increased urinary sodium excretion, and reduced renal vascular resistance only during ACE inhibition. These effects were abolished by ET_B_ blockade using BQ788 and nitric oxide synthase inhibition, whereas cyclooxygenase inhibition had no effect. These results showed that synergism between ET_A_ antagonism and ACE inhibition occurs via an ET_B_-mediated, nitric oxide–dependent, cyclooxygenase-independent mechanism. In patients with chronic kidney disease, TAK-044 beneficially reduced the mean arterial and systemic vascular resistance index and tended to increase renal plasma flow. TAK-044 had no effect on sodium or lithium clearance, or on the fractional excretion of sodium and lithium.^54^

Combining the results from a number of different studies has led to the proposal that antagonism of ET_B_ may be undesirable in conditions such as chronic renal failure, and therefore ET_A_-selective antagonists might be superior to mixed ET_A_/ET_B_ antagonists. This hypothesis was tested experimentally by comparing the action of BQ123 or BQ788 alone or in combination in hypertensive patients with chronic renal failure.^55^ Blocking the ET_A_ alone significantly reduced blood pressure in these patients. The magnitude of change was significantly higher than when ET_B_ also was blocked by BQ788. BQ788 alone caused the expected systemic and renal vasoconstriction, supporting the concept that ET_B_ maintain tonic renal vasodilatation in patients. BQ-123 infused alone increased renal blood flow and renal vascular resistance and reduced proteinuria, consistent with a renoprotective action. This effect was lost when ET_B_ were blocked by infusing both BQ788 and BQ123. There was no change in sodium excretion but this may have been the result of a comparatively small number of subjects.^55^

In a larger study of 22 patients with nondiabetic proteinuric chronic kidney disease, BQ-123 produced significant natriuresis, resulting from increased renal blood flow. In addition, ET_A_ antagonism reduced blood pressure and proteinuria, and, a new finding, decreased arterial stiffness.^56^ However, in diabetic patients with chronic kidney disease, avosentan (ET_A_-selective nonpeptide antagonist) was reported to be detrimental as a result of fluid overload.^57^

## ET-Receptor Blockade in Chronic Kidney Disease

Receptor antagonists have emerged as the only strategy in the clinic for blocking the unwanted actions of ET-1. To date, no alternative strategies, such as inhibitors of ET converting enzymes or combined endothelin-converting enzyme (ECE)/neutral endopeptidase (NEP) inhibitors, have been approved. Four compounds, bosentan, ambrisentan, sitaxentan, and macitentan, originally were approved for clinical use in pulmonary arterial hypertension (PAH) (Table 2).^11^ Sitaxentan, however, was withdrawn from clinical use in 2010^58^ after idiosyncratic hepatitis occurred resulting from acute liver failure, leading to death. PAH affects approximately 100,000 patients in the United States and Europe and currently there is no cure. The disease is characterized by constriction and remodeling of pulmonary vessels, with high blood pressure in the lungs. This leads to right heart failure, which is the ultimate cause of death. Interestingly, although ET_A_ are increased significantly in the failing right ventricle of patients with PAH^59^ and the failing left ventricle of patients with heart failure,^60^ clinical trails have failed to show a benefit in patients from the latter group.^12^ The reasons for this are unclear, but the action of ET antagonists on the vasculature may be more important in restoring the imbalance between ET-induced constriction and opposing vasodilatation of blood vessels.

In theory, the selectivity of antagonists should have pharmacologic and pathophysiological consequences. Selectively blocking smooth muscle ET_A_ would be expected to lead to vasodilatation and attenuate proliferation, migration, fibrosis, and hypertrophy. Endothelial ET_B_, particularly in kidney, lung, and liver, should continue to bind and remove ET-1 where it is overexpressed in pathophysiological conditions, as well as releasing vasodilators to mediate their antiproliferative and antithrombotic actions.

## How do we Define Antagonist-Receptor Selectivity?

These four antagonists represent a spectrum of selectivity ranging from bosentan, which is classified by the pharmaceutical company Actelion (Allschwil, Switzerland) as a mixed or balanced ET_A_/ET_B_ antagonist, to sitaxentan, the most ET_A_ selective. No consensus has emerged about the relative merits of mixed versus ET_A_-selective compounds in PAH.^61,62^ Some animal studies have suggested that selective ET_A_ antagonism that leaves ET_B_ unopposed and unblocked is beneficial,^63^ whereas other studies have shown mixed and ET_A_-selective antagonists have similar outcomes. The advantage of animal studies are that compounds can be compared head to head, but given differences in cell expression of subtypes these may not necessarily be informative of clinical studies in human beings. No clear-cut advantage of one over another has been reported for selective versus nonselective antagonism in PAH. However, in chronic kidney disease, the function and distribution of receptors suggests an ET_A_ antagonist would be preferable in blocking ET_A_-mediated constriction and proliferation but sparing endothelial cell vasodilatation, clearing ET from the plasma, and natriuresis.^64^

The selectivity of a ligand for two receptors usually is calculated by measuring the equilibrium dissociation constant (K_D_) for the two subtypes, in this case ET_A_ and ET_B_, to provide a ratio of selectivity^65,66^ in ligand-binding assays. There is no standardized method or general agreement among pharmaceutical companies to determine which compound should be classified as ET_A_ selective versus a mixed antagonist.^62^ Accurate information is essential in interpreting results from experiments in animal models and clinical trials as to whether the doses used are likely to result in a compound occupying only ET_A_ or both subtypes. We have proposed that ET_A_-selective compounds should have at least a 100-fold selectivity for the ET_A_ subtype whereas mixed antagonists should have less than 100-fold ET_A_ selectivity.^65^ The reason for this is that the degree of receptor occupancy achieved when an antagonist is administered in vivo or in vitro is proportional to the concentration and can be calculated from the affinity using the following formula: L*/(K_D_ + L*), where L* is the free ligand concentration and K_D_ is the affinity constant. For example, a compound that has an affinity measured in a ligand-binding assay of 1 nmol/L for ET_A_ but 100 nmol/L for ET_B_ would have 100-fold selectivity for ET_A_. By using this equation, at a concentration of 10 nmol/L, 90% of ET_A_ are calculated to be blocked but less than 10% of the ET_B_. Although this concentration can be achieved accurately under controlled in vitro conditions, 100-fold selectivity is likely to represent the minimum that can be used in vivo to achieve selective ET_A_ blockade. If the plasma concentration of this antagonist was increased to 100 nmol/L, 50% of ET_B_ then would be occupied. Compounds of greater than 1,000-fold selectivity are likely to be needed for clinical or in vivo studies to ensure ET_A_ selectivity is maintained.

As proof of principle, the effect of selective blockade was measured using TAK-044, a peptide antagonist with approximately 250-fold selectivity for the ET_A_ subtype over ET_B_ as measured by ligand binding in the human heart. A 30-mg infusion over 15 minutes of TAK-044 (providing a serum concentration of 2 nmol/L, calculated to block >95% of ET_A_ but <5% ET_B_) had no effect on the immunoreactive plasma concentrations of ET-1. However, after a higher dose of 750 mg TAK-044 (providing a serum concentration of 80 nmol/L, calculated to block >99% of ET_A_ and >75% ET_B_), the immunoreactive plasma ET-1 concentrations were increased more than three-fold over basal levels. Importantly, the concentrations of the ET-1 precursor or C-terminal fragment of big endothelin-1 were unchanged, indicating that the increase in ET-1 in the plasma was unlikely to be the result of increased synthesis or release. The most likely sources of endothelin contributing to the observed increase were displacement of receptor-bound peptide and a reduction in plasma clearance mediated by ET_B_.^45^

## Does Selectivity Matter in Chronic Kidney Disease?

Blocking ET_B_ clearly results in a significant increase in circulating plasma ET-1 levels. However, with a mixed antagonist, this increase is unlikely to be important because the vasoconstrictor ET_A_ also is blocked. Side effects including headache, nausea, and nasal congestion have, to a certain extent, been reported for ET_A_/ET_B_ mixed antagonists and with ET_A_-selective compounds. For ET_A_-selective compound such as ambrisentan, nasal congestion and peripheral edema are more prevalent but they have less of the hepatic effects such as an increase in liver enzyme levels that require liver function tests and drug–drug interactions that are associated with mixed antagonists such as bosentan.^67^ Studies in mice selectively knocking out ET_A_ from the nephron or collecting duct did not show ET_A_ antagonist-induced fluid retention and this was attenuated where ET_A_ smooth muscle had been deleted, suggesting the mechanism is a direct action on collecting duct receptors and partially within the vasculature.^68^

Liver toxicity has been a significant problem with bosentan but its mechanism of action has been proposed to be independent of ET receptors and is thought to occur by inhibiting the bile salt export pump leading to accumulation of cytotoxic bile salts, resulting in hepatocellular damage. In contrast, macitentan is thought to enter the liver via passive diffusion and not by active uptake.^69^ As a result, macitentan has been reported to have a better safety profile compared with bosentan for hepatic toxicity. This is an important consideration in patients with renal or hepatic disease.^70^ Bosentan is a competitive antagonist of ET_A_ and ET_B_ of the sulfonamide class,^71^ with a comparatively short half-life and good bioavailability. Bosentan, as with other dual antagonists, tends to have lower rates of fluid retention and edema when used clinically. Although bosentan has been shown to be effective in animal models of renal disease, the compound has not been evaluated in detailed clinical trials involving renal patients. Ambrisentan represents the second chemical class,^71^ is less ET_A_ selective than sitaxentan, but has good bioavailability and a long half-life. However, again clinical studies have not been reported in chronic kidney disease. Key clinical studies have been performed using sitaxentan,^71^ the most ET_A_-selective antagonist that largely supports the hypothesis of selective ET blockade. A randomized, double-blind, three-way, cross-over study of patients with proteinuric chronic kidney disease compared sitaxentan and nifedipine with placebo for proteinuria, blood pressure, and arterial stiffness. As expected, plasma levels of ET-1 were unchanged during sitaxentan treatment, indicating that ET-1 continued to be cleared from the circulation but urinary ET-1 levels were decreased. Blood pressure, arterial stiffness, and proteinuria also were reduced significantly over 6 weeks. Intriguingly, asymmetric dimethylarginine, an endogenous inhibitor of nitric oxide synthases that is considered an independent marker of disease progression, was increased in these patients, supporting the concept that sparing ET_B_ receptors from blockade with sitaxentan treatment would modulate the nitric oxide pathway. Importantly, these effects were seen in patients already receiving optimal treatment with ACE inhibitors and angiotensin blockers. A related study also found an increase in nocturnal dipping in blood pressure with sitaxentan.^72^ The results suggested that ET_A_ antagonism had additional longer-term renoprotective effects in patients with chronic kidney disease.^73^

## ET Antagonist for the Future: Macitentan and Atresentan

Macitentan is an insurmountable antagonist, resulting from structure-activity studies to improve the efficacy and tolerability of bosentan, and gained approval in the United States in 2013 for the treatment of PAH. Actelion describes the compound as a dual antagonist, but on the basis of their own data measuring inhibition of [^125^I]–ET-1 binding to human-expressed receptors it displays approximately 800-fold selectivity for the ET_A_ subtype.^74^ However, plasma ET-1 concentrations were increased significantly (two-fold at the highest dose tested), suggesting blocking of ET_B_ occurs at the dose used.^75^ A metabolite of macitentan, ACT-132577, is active pharmacologically, albeit with a lower potency, but reaches higher plasma concentrations and has a longer half-life than macitentan.^76–79^ Key pharmacologic parameters suggest macitentan will have the potential for greater efficacy and safety than bosentan. Macitentan has a much longer receptor occupancy (17 minutes compared with 70 seconds for bosentan), probably as a result of interaction with different amino acid residues in the ET receptors and is an order of magnitude more potent than bosentan, measured by in vitro assays. Pharmacokinetic benefits include fewer interactions with other drugs, with no requirement to alter doses in patients with renal (or hepatic) impairment. Crucially, the compound has improved hepatic safety and reduced edema/fluid retention compared with bosentan.^79,80^ A number of clinical trials are actively recruiting, however, these do not yet include chronic kidney disease patients.^81^

Clinical trials also recently were reported on an investigational ET_A_-selective antagonist: atrasentan (ABT 627).^82^ The aim of the double-blind study performed in parallel at two centers was to determine whether albuminuria was reduced further when atrasentan was administered at two different doses, with inhibitors of the renin-angiotensin system, to patients with type 2 diabetic nephropathy. Atrasentan reduced albuminuria at both doses tested, and reduced blood pressure, cholesterol, and triglyceride levels, with unwanted side effects being more manageable at the lower dose. These promising results lead to the initiation of a phase 3 multicenter trial (Study Of Diabetic Nephropathy With Atrasentan^83^) with 4,000 patients.

## Perspectives

After more than 25 years since the discovery of ET, the peptide remains the most powerful and long-lasting constrictor of the human vasculature including the kidney described to date. In pathophysiological conditions, ET-1 contributes to vascular remodeling, proliferation of mesangial cells, and extracellular matrix production mainly through binding to ET_A_. Beneficial actions of ET-1 on sodium and water regulation are mainly ET_B_-mediated. These findings suggest an ET_A_-selective antagonist would have a therapeutic advantage over a mixed antagonist in renal disease, and indeed the small number of acute studies directly comparing the peptide antagonists BQ123 versus BQ788 suggest ET_A_ blockade, sparing ET_B_, may be beneficial. However, this is balanced by the possible greater prevalence of side effects such as edema reported for small-molecule, orally active ET_A_ antagonists compared with mixed antagonists, although the latter also have their limitations because of liver toxicity. In addition, head-to-head studies in patients comparing orally active ET_A_ antagonists with mixed antagonists have not been performed. The renal ET system remains a compelling target: will new therapies be clinically relevant in the future? This question may be answered by the next generation of ET antagonists.

## Figures and Tables

**Table 1:** Key ET Agonists and Antagonists Used in Research and Clinical Studies (Including Only Those Approved for Clinical Use in Clinical Studies), and the Functional Role of Renal ET Receptors

	ET_A_	ET_A_/ET_A_	ET_B_
Potency	ET-1 = ET-2 > ET-3		ET-1 = ET-2 = ET-3
Peptide agonists			Sarafotoxin S6C
			BQ3020
			IRL1620
Peptide antagonists	BQ123		BQ788
	FR139317		
	Tak-044		
Clinically approved antagonists			
Selective	Sitaxentan		
	Ambrisentan		
Mixed		Bosentan	
		Macitentan	


Renal Function	ET_A_	ET_B_
Vasculature	Cortical vasoconstriction	Medullary vasodilatation
	Conduit vessel vasoconstriction	Conduit vessel vasodilatation
	Afferent arteriolar constriction	Afferent arteriolar dilatation
	Efferent arteriolar constriction	Efferent arteriolar dilatation
Glomerulus	Mesangial cell contraction	
	Mesangial cell proliferation	
	Podocyte injury	
Epithelium		Endothelial function not known
Inner medullary	Extracellular matrix accumulation	Natriuresis
collecting duct	Interstitial fibrosis	
	Possible natriuresis	

**Table 2:** Structure and Pharmacokinetic Properties of ET-Receptor Antagonists in Clinical Use

	Bosentan	Macitentan	Active metabolite of macitentan	Ambrisentan	Sitaxentan (withdrawn from clinical use in 2010)
Trade name	Tracleer (Actelion, Allschwil, Switzerland)	Opsumit (Actelion)		Letairis, Volibris (Gilead, Foster City, California)	Thelin (Pfizer, Groton, Connecticut)
Other names	Ro47-0203	ACT-064992	ACT-132577	LU-208075	TBC-11251
Chemical name	Benzenesulfonamide	Sulfamide	Sulfamide	Benzenepropanoic acid	3-Thiophenesulfonamide
Structure					
ET plasma levels after administration	↑↑	↑↑	-	↑	↓
Bioavailability	~50%	Not reported	Not reported	High	70%-100%
Time to maximum plasma concentration	3-5	4-12	30	1.7-3.3	1-4
Terminal half life (hours)	5.4	16	40.2-65.6	15	10
Excretion in urine (%)	<3	Not detected	Not detected	Low	50-60
